# Clinicopathologic and Molecular Characteristics of High-Grade Appendiceal Mucinous Neoplasms

**DOI:** 10.1245/s10434-025-18672-0

**Published:** 2025-11-12

**Authors:** Ashlee N. Seldomridge, Michael G. White, Christopher Scally, Richard E. Royal, Mark Routbort, JBryan Iorgulescu, Michael J. Overman, Sun Mi Lee, Wai Chin Foo, Melissa W. Taggart, Yun Song, Paul F. Mansfield, Keith F. Fournier, John Paul Shen, Beth A. Helmink

**Affiliations:** 1 Department of Surgical Oncology, Division of Surgery, The University of Texas MD Anderson Cancer Center, Houston, TX USA; 2 Department of Colon and Rectal Surgery, Division of Surgery, The University of Texas MD Anderson Cancer Center, Houston, TX USA; 3 Division of Surgical Oncology, Department of Surgery, Maine Medical Center, Portland, ME USA; 4 Molecular Diagnostics Laboratory, Department of Hematopathology, Division of Pathology-Lab Medicine, The University of Texas MD Anderson Cancer Center, Houston, TX USA; 5 Department of Gastrointestinal Medical Oncology, Division of Cancer Medicine, The University of Texas MD Anderson Cancer Center, Houston, TX USA; 6 Department of Anatomic Pathology, Division of Pathology-Lab Medicine, The University of Texas MD Anderson Cancer Center, Houston, TX USA

**Keywords:** High-grade appendiceal mucinous neoplasm, HAMN, Cytoreduction, HIPEC

## Abstract

**Background:**

Although 2016 consensus guidelines defined high-grade appendiceal mucinous neoplasms (HAMNs) as a distinct histologic entity, their clinical course and optimal management remain poorly characterized. Comparative data with low-grade appendiceal mucinous neoplasms (LAMNs) are limited. This study aims to detail the clinical management and outcomes of patients with HAMN and those with LAMN.

**Methods:**

This retrospective cohort study was conducted at a large academic cancer center. An internal database was queried to identify patients with LAMN or HAMN who underwent appendectomy between 2016 and 2024. Kaplan–Meier survival analyses were used to assess the impact of primary tumor type and peritoneal disease (PD) on recurrence-free survival (RFS) and overall survival (OS).

**Results:**

Of the 375 patients, 276 (73.6%) had LAMN and 99 (26.4%) had HAMN. Localized disease was most common (187 patients [49.9%]). Acellular mucin, low-grade mucinous carcinoma peritonei (LGMCP), and high-grade mucinous carcinoma peritonei (HGMCP) were observed in 14.9, 25.3, and 9.9% of patients, respectively. HAMNs were associated with increased PD (69.7% vs 43.1%, *P* < 0.001) and HGMCP (19.2% vs 6.5%, *P* < 0.001). Patients with localized disease or acellular mucin experienced almost no recurrences (99.2%). Patients with HAMN trended towards worse 5 year RFS (80.7% vs 90.4%, *P *= 0.083). OS was similar (91.1% vs LAMN 93.4%, *P *= 0.23).

**Conclusions:**

This is the largest cohort of patients with HAMN characterized to date. HAMNs are associated with more frequent and higher-grade PD than are LAMNs. The presence and type of PD influence both RFS and OS for patients with AMN. Overall, there is a trend towards decreased RFS in patients with HAMN than with LAMN, but OS was similar.

**Supplementary Information:**

The online version contains supplementary material available at 10.1245/s10434-025-18672-0.

Appendiceal mucinous neoplasms (AMNs) are uncommon epithelial tumors of the appendix distinguished by abundant mucin production and characteristic pushing, rather than infiltrative or destructive, invasion of the appendiceal wall.^1,2^ Mucin accumulation and perforation can lead to dissemination of mucin (M1a) and/or tumor (M1b) throughout the peritoneal cavity, a clinical syndrome called pseudomyxoma peritonei for which the standard of care is cytoreductive surgery (CRS) and hyperthermic intraperitoneal chemotherapy (HIPEC).^3–5^ Notably, peritoneal disease (PD) can be low or high grade (low-grade mucinous carcinoma peritonei [LGMCP] vs high-grade mucinous carcinoma peritonei [HGMCP]), and clinical outcomes following CRS-HIPEC largely depend on both tumor grade and disease extent. However, not all AMNs lead to perforation and/or peritoneal dissemination of disease.

Despite the characteristic lack of infiltrative invasion, AMNs can contain areas of high-grade cytologic atypia. There have been significant inconsistencies in the histopathologic classification of such AMNs with high-grade cytologic atypia, with some even using the seemingly contradictory term, non-invasive appendiceal adenocarcinoma, to describe these lesions.^1,2,6,7^ In 2016, the Peritoneal Surface Oncology Group International (PSOGI) reached a consensus on classifications for AMNs, recommending the terms low-grade appendiceal mucinous neoplasm (LAMN) and high-grade appendiceal mucinous neoplasm (HAMN) to reflect the grade of cytologic atypia identified within the AMN.^8^

As this is a relatively new diagnostic entity, the clinical behavior of HAMN as compared with LAMN is not clearly defined, and understanding has been driven by limited case series and small cohort studies of up to 38 patients (Supplemental Table S1).^9–24^ Some of these reports suggest a more aggressive phenotype for HAMN than for LAMN,^2,7,24^ whereas others found outcomes similar to those with LAMN,^11,17,18,25^ particularly in the absence of cellular PD. Interestingly, HAMN is currently classified by the World Health Organization (WHO) as G2 or moderately differentiated and shares the same TNM staging as appendiceal adenocarcinoma in the American Joint Committee on Cancer (AJCC) 9^th^ edition guidelines.^5,26^ Further, clinical practice guidelines are inconsistent for HAMN (Supplemental Table S2).^5,27–32^

The objective of this study was to assess the clinicopathologic characteristics, risk of peritoneal dissemination, surgical management, and long-term outcomes of patients diagnosed with HAMN versus LAMN. This represents the largest cohort of patients with HAMNs described to date.

## Methods

### Study Design

The MD Anderson Cancer Center (MDACC) institutional review board approved the collection of clinicopathologic information under the institutional review board protocol. The Foundry software system^33,34^ (Palantir, Denver, CO, USA), a platform that assists in data extraction from the electronic health record, was used to query the MDACC internal patient database to identify all patients who had (1) a diagnosis of AMN, (2) appendectomy between 2016 and 2024, and (3) review of primary tumor pathology by the MDACC Department of Pathology for inclusion in this retrospective cohort study. The data collection cutoff point was January 15, 2025. Most clinical data were collected by manual chart review; all extracted data were manually verified by study authors. This study followed the Strengthening the Reporting of Observational Studies in Epidemiology (STROBE) guidelines for cohort studies.^35^

### Variable Definitions

Standard patient demographic data and tumor characteristics were recorded. “Appendectomy” included partial cecectomy and completion appendectomy (resection of appendiceal stump). “Partial colectomy” included right hemicolectomy (RHC) and ileocecectomy. Complete cytoreduction was defined as completeness of cytoreduction (CCR) scores 0–1. “Incomplete CRS” included CCR 2–3 cytoreduction with or without HIPEC as well as other procedures, including total abdominal hysterectomy, bilateral salpingo-oophorectomy, omentectomy, resection of pelvic mass, and low anterior resection resulting in partial removal of tumor. “Definitive surgery” is defined as the first surgery to result in “absence of disease” or CCR 0–1 resection. Pathologic diagnosis was determined by a team of expert gastrointestinal pathologists (including S.M.L, M.W.T., and W.C.F.). Review of the primary tumor at MDACC was necessary to mitigate variability between institutions and pathologists. T stage was assigned according to AJCC criteria. Perforated tumors with acellular or cellular mucin limited to the muscularis propria and fibrous wall, but focally present on the serosa with associated mesothelial reaction, posed classification challenges. In such cases, the distinction between luminal contamination, carry-over artifact, and true fibrotic healing from prior appendiceal perforation could not be determined with certainty. Similarly, clear guidance for perforated tumors without evidence for serosal mucin is lacking. Because AJCC provides no specific recommendations for these scenarios, we classified all perforated tumors as T4a. PD was classified according to the PSOGI guidelines. LGMCP included peritoneal implants characterized as cellular mucin, well-differentiated, and well-to-moderately differentiated adenocarcinoma (AJCC G1). HGMCP included peritoneal implants characterized as moderately differentiated (AJCC G2), moderately-to-poorly differentiated, and poorly differentiated adenocarcinoma (AJCC G3). For our cohort, the presence of signet ring cells (SRCs) was included within HGMCP. For the purpose of this paper, we included the following categories, acellular mucin, LGMCP, and HGMCP ± SRCs; for patients with heterogeneity within their PD, the highest grade present was used for classification. Preoperative serum tumor markers, defined as those measured directly before CRS-HIPEC, were recorded. Elevated levels of carcinoembryonic antigen (CEA), carbohydrate antigen 19-9 (CA 19-9), and cancer antigen 125 (CA 125) were defined as > 5.5 ng/mL, > 35 U/mL, and > 38 U/mL, respectively. Survival outcomes data were collected at the time of last patient follow-up. Overall survival (OS) was defined as the time from initial diagnosis until date of last follow-up or death, and recurrence-free survival (RFS) was defined as the time from “definitive surgery” to peritoneal recurrence, censoring at the date of last follow-up or death in the absence of recurrence. Deaths without recurrence were censored for the RFS analysis.

### Statistical Analyses

Data were analyzed using R version 4.4.1.^36^ Next-generation sequencing was performed at MD Anderson’s molecular diagnostics laboratory, which is Clinical Laboratory Improvement Amendments certified. Molecular data were also collected from external genomic testing, and gene panels were compared to allow for combined analysis. All mutation data were deidentified and converted to mutation annotation format (MAF) for data visualization and analysis using the Bioconductor R package, Maftools.^37^ The Waterfall function within the GenVisR package was used to visualize the somatic mutation distribution.^38^ Clinicopathologic characteristics were summarized according to AMN. Medians with interquartile ranges (IQRs) were used for continuous variables, and frequencies with proportions were used for categorical variables. The Kruskal–Wallis test was used to analyze differences in median. Chi-squared or Fisher’s exact tests were used to assess differences in proportions. Survival outcomes were summarized by the Kaplan–Meier method, and the log-rank test was used to assess differences between groups.

## Results

### Patient Characteristics

**Fig. 1:**
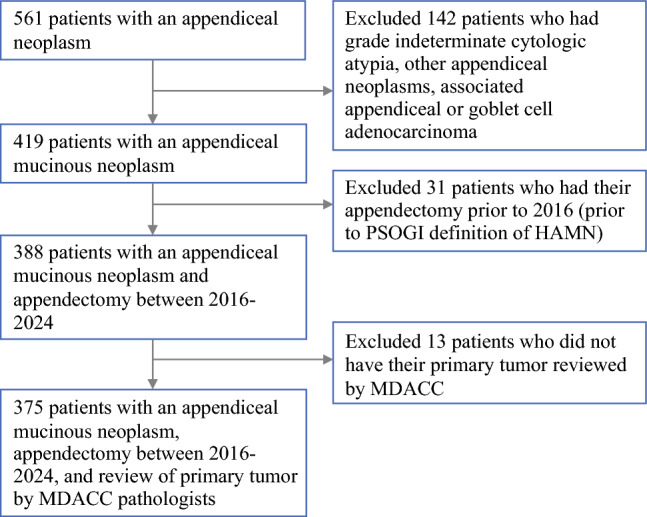
Flow diagram of patient selection and exclusion criteria. HAMN, high-grade appendiceal mucinous neoplasm; MDACC, MD Anderson Cancer Center; PSOGI, Peritoneal Surface Oncology Group International

A total of 375 patients were included (Fig. 1). Of these, 276 (73.6%) had LAMN and 99 (26.4%) had HAMN (Table 1). The median age was 57 years (IQR 46–66), and 249 patients (66.4%) were female. The median follow-up for the entire cohort was 37.4 months. The cohort of patients with HAMN had a higher proportion identifying as Black or African American (N = 9 [9.1%]) than did the LAMN cohort (N = 10 [3.6%]) or as belonging to Other racial groups (N = 14 [14.1%] vs. N = 24 [8.7%], *P *= 0.02).

**Table 1:** Patient characteristics according to primary tumor type

Characteristic	Overall N = 375	LAMN N = 276	HAMN N = 99	*P*-value^a^
Age, years	57 (46–66)	57 (46–65.5)	57 (47–66)	0.54
Sex, female	249 (66.4)	189 (68.5)	60 (60.6)	0.15
Race				0.024
White	318 (84.8)	242 (87.7)	76 (76.8)	
Black	19 (5.1)	10 (3.6)	9 (9.1)	
Other	38 (10.1)	24 (8.7)	14 (14.1)	
Ethnicity	53 (14.1)	40 (14.5)	13 (13.1)	0.74
Perforation	124 (33.1)	82 (29.7)	42 (42.4)	0.021
PCI	19 (14-28)	20 (14-26)	19 (14-33)	0.15
Peritoneal disease^b^				< 0.001
No disease	187 (49.9)	157 (56.9)	30 (30.3)	
Acellular mucin	56 (14.9)	32 (11.6)	24 (24.2)	
LGMCP	95 (25.3)	69 (25.0)	26 (26.3)	
HGMCP ± SRC	37 (9.9)	18 (6.5)	19 (19.2)	
Surgical management				< 0.001
Appendectomy^c^	160 (42.7)	129 (46.7)	31 (31.3)	
Partial colectomy^d^	41 (10.9)	35 (12.7)	6 (6.1)	
Complete CRS^e^	30 (8.0)	17 (6.2)	13 (13.1)	
Complete CRS + HIPEC^e^	115 (30.7)	81 (29.3)	34 (34.3)	
Incomplete CRS ± HIPEC^f^	29 (7.7)	14 (5.1)	15 (15.2)	
Path T stage				< 0.001
Tis(LAMN)/T1/T2	63 (16.8)	54 (19.6)	9 (9.1)	
T3	60 (16)	52 (18.8)	8 (8.1)	
T4a	227 (60.5)	153 (55.4)	74 (74.7)	
T4b	19 (5.1)	11 (4)	8 (8.1)	
TX	6 (1.6)	6 (2.2)	0 (0)	
CEA > 5.5 ng/mL	54 (43.2)	33 (39.8)	21 (50)	0.27
CA19-9 > 35 U/mL	27 (22)	16 (19.5)	11 (26.8)	0.36
CA125 >38 U/mL	40 (32.5)	24 (29.3)	16 (39.0)	0.28

Data are presented as median (Q1–Q3) or n (%) unless otherwise indicated.

^a^ Wilcoxon rank sum test; Pearson’s chi-squared test; Fisher’s exact test

^b^ Highest grade of peritoneal disease

^c^ Appendectomy included partial cecectomy and completion appendectomy

^d^ Partial colectomy included right hemicolectomy and ileocecectomy

^e^ Complete CRS defined as CCR score of 0–1

^f^ Incomplete CRS ± HIPEC included CCR 2–3 CRS or debulking surgeries

*CA 125* carbohydrate antigen 125; *CA 19-9* carbohydrate antigen 19-9; *CEA* carcinoembryonic antigen; *CRS* cytoreductive surgery; *HAMN* high-grade appendiceal mucinous neoplasm; *HGMCP ± SRCs* high-grade mucinous carcinoma peritonei with or without signet ring cells; *HIPEC* hyperthermic intraperitoneal chemotherapy; *LAMN* low-grade appendiceal mucinous neoplasm; *LGMCP* low-grade mucinous carcinoma peritonei; *PCI* peritoneal carcinomatosis index

#### HAMNs Demonstrate More Advanced Pathologic T Stage and Higher-Grade Peritoneal Dissemination

Pathologic T stage differed significantly between groups: 74 patients (74.7%) with HAMN had pT4a disease compared with 153 patients (55.4%) with LAMN (*P *< 0.001). The overall perforation rate of the primary lesion was 33.1% (N = 124), with a higher incidence with HAMN than with LAMN (N = 42 [42.4%] vs. N = 82 [29.7%], *P *= 0.021).

**Fig. 2:**
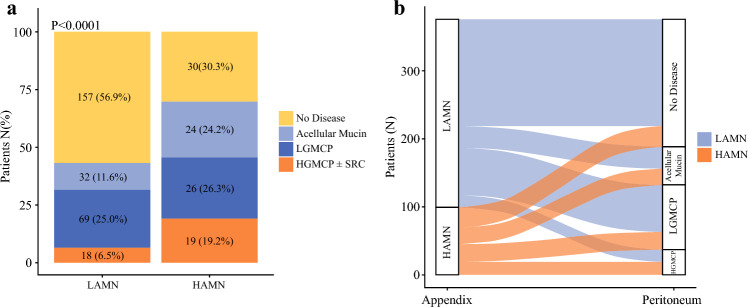
**a** Stacked bar plot showing the distribution of peritoneal disease (PD) among 375 patients with low-grade appendiceal mucinous neoplasm (LAMN) or high-grade appendiceal mucinous neoplasm (HAMN). PD was more frequent in those with HAMN (69.7%) than in those with LAMN (43.1%, *P *< 0.001). **b** Alluvial diagram illustrating the relationship between appendiceal mucinous neoplasm histologic grade and the type of PD. HAMNs were more commonly associated with higher-grade PD, and LAMNs were more often associated with low-grade mucinous carcinoma peritonei (LGMCP). *HGMCP* high-grade mucinous carcinoma peritonei; *LGMCP* low-grade mucinous carcinoma peritonei; *SRC* signet ring cells

Half of all patients had disease confined to the appendix without evidence of peritoneal dissemination. Among patients with peritoneal dissemination, 56 (29.8%) had PD consisting of acellular mucin, 95 (50.5%) had LGMCP, and 37 (19.7%) had HGMCP ± SRC. PD was significantly more common with HAMN (N = 69 [69.7%]) than with LAMN (N = 119 [43.1%], *P *< 0.001) (Fig. 2). In nearly all cases, PD was present at the time of initial clinical presentation, described here as synchronous metastases; only two patients (0.5%) presented without PD and later went on to develop it. HAMN generated LGMCP slightly more frequently than HGMCP ± SRC (N = 26 [26.3%] vs. N = 19 [19.2%]). However, the rate of HGMCP ± SRC was nearly three times higher in HAMN as in LAMN (N = 19 [19.2%] vs. N = 18 [6.5%], *P *< 0.001). Collectively, these findings demonstrate that HAMNs are more often associated with PD, and this is more often HGMCP than it is with LAMNs.

### Surgical Management of AMNs by Extent of PD

Surgical management of AMNs is dictated by the presence or absence of PD. In total, 346 patients (92.3%) were rendered disease-free by surgery. For those with localized disease, 150 (80.2%) had an appendectomy, which could include partial cecectomy, and 37 (19.8%) had a partial colectomy, which included ileocecectomy or formal RHC. A total of 10 patients underwent ileocecectomy, all of whom had LAMN (6.4%), and a total of 27 patients underwent formal RHC (LAMN N = 23 [14.6%] and HAMN N = 4 [13.3%]). No lymph node metastases were identified in either group, with a median of 15 lymph nodes (IQR 3–26) harvested. Notably, the reason for colectomy was not uniformly reported.

Of the patients with PD, 30 (16%) underwent complete CRS (CCR 0–1) and 115 (61.2%) underwent complete CRS with HIPEC. Those with PD who did not undergo complete CRS ± HIPEC had incomplete CRS or incomplete CRS with HIPEC (CCR 2–3 CRS or other debulking surgeries), which did not render them disease free. Patients with HAMN had a higher rate of incomplete CRS ± HIPEC (N = 15 [21.7%] vs. LAMN N = 14 [11.8%], *P *= 0.11), particularly those with HGMCP ± SRC (N = 8 [53.3%] vs. N = 3 [21.4%] for LAMN, *P *= 0.034).

Of those with PD undergoing CRS with HIPEC, the median peritoneal carcinomatosis index (PCI) score was 20 (IQR 14–28). Elevated PCI ( > 20) was similar between LAMN and HAMN (N = 38 [45.8%] vs. N = 21 [48.8%], *P *= 0.75). A complete resection (CCR 0–1) was achieved in 88.5% of all patients who underwent CRS with HIPEC (LAMN 81 [93.1%] vs. HAMN 34 [79.1%], *P*=0.037); of those who underwent CRS with HIPEC with curative (non-palliative) intent, 99.1% achieved CCR 0–1. Elevated preoperative serum tumor markers were less frequent in patients with LAMN than in those with HAMN, although this was not statistically significant (CA 19-9: 19.5% vs. 26.8%, *P *= 0.36; CEA: 39.8% vs. 50%, *P *= 0.27; CA 125: 29.3% vs. 39%, *P *= 0.28, respectively). The proportion of patients with elevation in any tumor marker was 18% (N = 49) for LAMN and 25% (N = 25) for HAMN (*P *= 0.11).

### TP53 Mutations Identified within HAMN

**Fig. 3:**
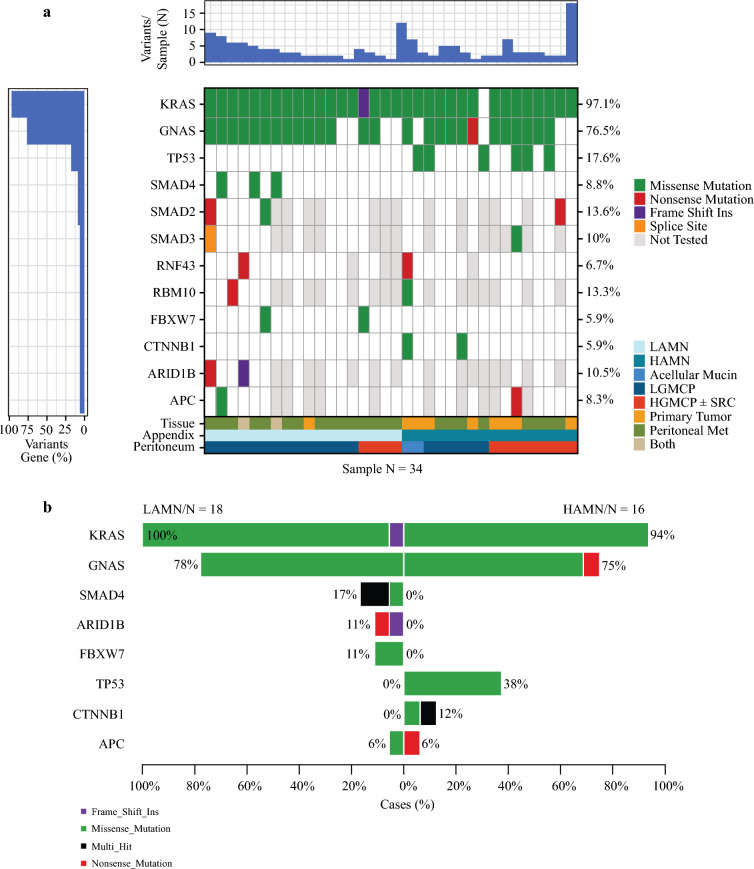
Somatic mutations in tumor samples (N = 34) from patients with low-grade appendiceal mucinous neoplasm (LAMN) or high-grade appendiceal mucinous neoplasm (HAMN). Each patient corresponds to one tissue sample. **a** Waterfall plot. The top bar plot shows the total number of mutations per sample. The left bar plot indicates the proportion of samples harboring mutations in each gene (not adjusted for gene panel). The central heatmap depicts gene-level variants, limited to genes mutated in at least 5% of samples (N = 12), with samples ordered by clinical metadata displayed at the bottom. **b** Comparison of relative proportions of LAMN and HAMN samples among the top eight most frequently co-occurring genes. *HGMCP* high-grade mucinous carcinoma peritonei; *LGMCP* low-grade mucinous carcinoma peritonei; *SRC* signet ring cells

Tumor somatic mutations were analyzed to understand their contribution to observed clinical behavior. High-throughput next-generation sequencing data, obtained for clinical management, was reviewed for detection of variants/mutations in cancer-related genes. A total of 34 patients (LAMN N = 18 [52.9%], HAMN N = 16 [47.1%]) had molecular data available. The tissue samples used for molecular analyses included 23 peritoneal metastatic deposits (67.6%), nine primary tumors (26.5%), and two primary and metastatic samples (5.9%) (Fig. 3A). *KRAS* (N = 33 [97.1%]) and *GNAS* (N = 26 [76.5%]) mutations were the most common, followed by *TP53* (N = 6 [17.6%]) (Fig. 3A). Interestingly, *TP53* mutations were only identified in patients with HAMN; this included one patient with acellular mucin only, two patients with LGMCP, and three with HGMCP (Fig. 3B). Amino acid changes and additional mutations (for all samples, including those with < 5% variants detected) are provided in Supplemental Table S3.

### Influence of Primary Tumor and Type of PD on RFS

**Fig. 4:**
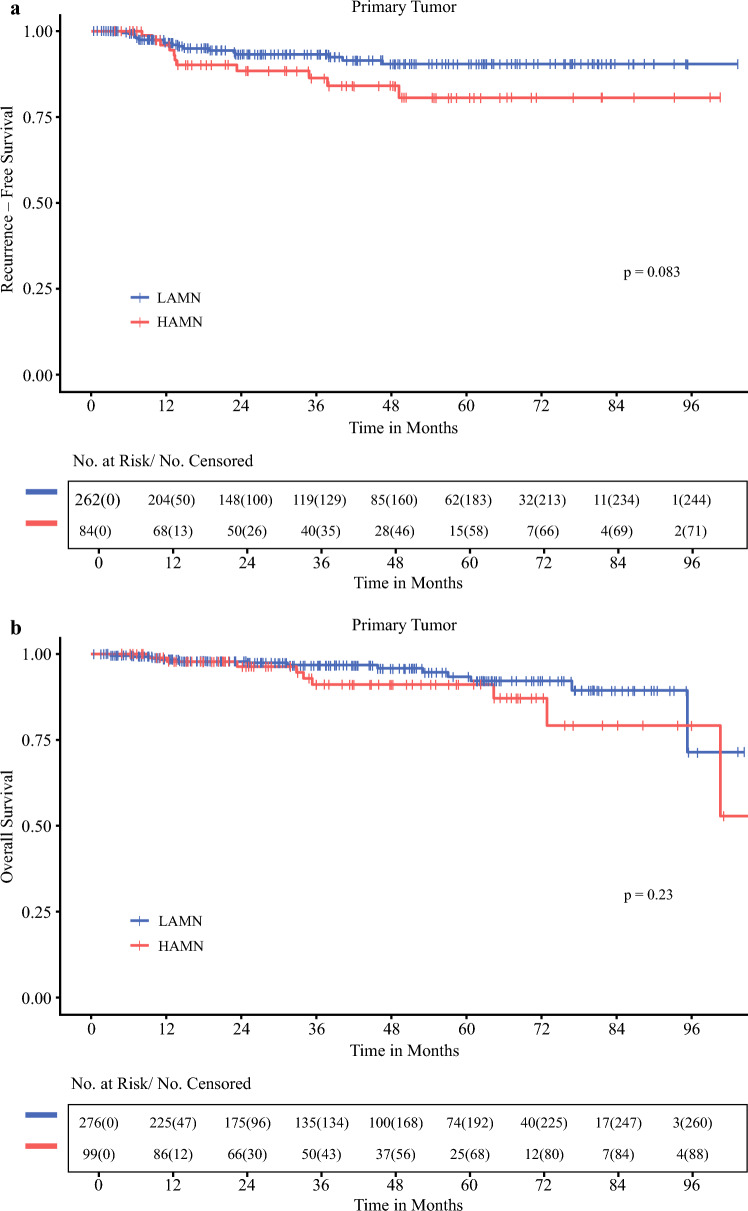
Kaplan–Meier survival analyses according to primary tumor type: **a** Recurrence-free survival and **b** Overall survival comparing low-grade appendiceal mucinous neoplasm (LAMN) and high-grade appendiceal mucinous neoplasm (HAMN)

The median duration of RFS was not reached (NR) for the entire cohort, at a median follow-up of 37.4 months. The 5-year RFS rate was 90.4% (95% CI 85.9–95.1) for all patients with LAMN and 80.7% (95% CI 70.4–92.5) for all patients with HAMN (*P *= 0.083) (Fig. 4A).

RFS was influenced by the presence and type of PD for all patients with AMN. The 5 year RFS rate for localized AMN as well as those with PD limited to acellular mucin was 98.8%. The 5 year RFS rate for patients with AMN and LGMCP or HGMCP was 67.1 and 46.9%, respectively (Supplemental Fig. S1).

**Fig. 5:**
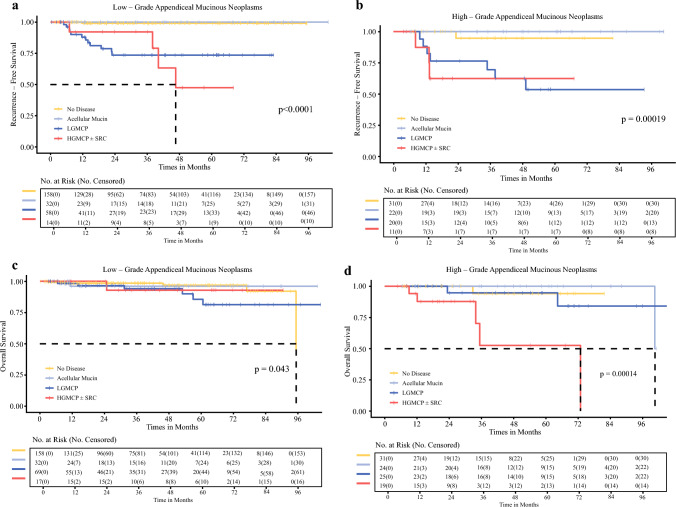
Kaplan–Meier survival analyses in patients with high-grade appendiceal mucinous neoplasm (HAMN) and low-grade appendiceal mucinous neoplasm (LAMN), stratified by peritoneal disease. Recurrence-free survival of (**a**) LAMN and (**b**) HAMN by peritoneal disease. Overall survival of (**c**) LAMN and (**d**) HAMN by peritoneal disease. HGMCP, high-grade mucinous carcinoma peritonei; LGMCP, low-grade mucinous carcinoma peritonei; SRC, signet ring cells

Of patients with localized LAMN, as well as those with LAMN and PD limited to acellular mucin, only one recurrence was noted (Fig. 5A). Similarly, of all patients with localized HAMN, as well as those with HAMN and PD limited to acellular mucin, we observed only one recurrence (Fig. 5B).

Analyses of RFS in patients with LGMCP and HGMCP ± SRC broken down by primary tumor type was limited by low patient numbers (LAMN N = 58 and HAMN N = 20 for LGMCP, and LAMN N = 14 and HAMN N = 11 for HGMCP). The median duration of RFS for patients with LAMN and HAMN with LGMCP was NR. The 5 year RFS rate for patients with LGMCP was 73.6% (95% CI 61.7–87.8) for patients with LAMN (Fig. 5A) and 53.6% (95% CI 32.9–87.4) for patients with HAMN (Fig. 5B) (*P *= 0.38, Supplemental Fig. S2). The median duration of RFS of patients with HGMCP ± SRC was 46.6 months (95% CI 40.2–NR) for patients with LAMN (Fig. 5A) and NR (95% CI 13.4–NR) for patients with HAMN (Fig. 5B). The 5 year RFS rate for patients with HGMCP was 47.5% (95% CI 21.5–100) for patients with LAMN (Fig. 5A) and 62.5% (95% CI 36.5–100) for patients with HAMN (*P *= 0.4, Supplemental Fig. S3).

### Influence of Primary Tumor and Type of PD on OS

There was no difference in the 5 year OS rates between patients with LAMN (93.4% [95% CI 89.1–97.9]) or HAMN (91.1% [95% CI 84.4–98.3], *P *= 0.23) (Fig. 4B).

OS was associated with the presence and type of PD for all patients with AMN. The 5 year OS rate for patients with AMN with localized disease or PD limited to acellular mucin was 96.8%. The 5 year OS rates for patients with AMN and LGMCP or HGMCP were 88.9 and 77.5%, respectively.

The median duration of OS for patients with LAMN and HAMN with LGMCP was NR. The 5 year OS rates for patients with localized LAMN and HAMN were 96.9 and 94.1%, respectively. The 5 year OS rates for patients with LAMN and HAMN with acellular mucin were 96.2 and 100%, respectively. For patients with LAMN and LGMCP, the 5 year OS rate was 85.7% (95% CI 74–99.3) (Fig. 5C) compared with 94.7% (95% CI 85.2–100) for patients with HAMN and LGMCP (Fig. 5D) (*P *= 0.66, Supplemental Fig. S4). For patients with LAMN and HGMCP ± SRC, the 5 year OS rate was 92.9% (95% CI 80.3–100) (Fig. 5C) compared with 52.7% (95% CI 25.2–100) for those with HAMN and HGMCP ± SRC (Fig. 5D) (P = 0.023, Supplemental Fig. S5).

## Discussion

To date, this is the largest published clinical cohort of patients with HAMNs. As this is a relatively newly categorized clinicopathologic entity, these data represent an important cohort of patients with several key implications.

First, as in patients with LAMN, margin-negative appendectomy appears to be adequate treatment for patients with HAMN confined to the appendix. We observed very few recurrences in patients presenting with HAMN alone after appendectomy (even with evidence of perforation). Further, among those who did undergo an RHC for HAMN without peritoneal metastases, no nodal metastases were identified. This agrees with the recently published RENAPE study in which 34 patients with HAMN underwent RHC with no nodal metastases identified.^24^

We observed the full spectrum of patients with PD with both LAMNs and HAMNs, including acellular mucin, LGMCP, and HGMCP ± SRC. Rates of discordance (i.e., LAMN with HGMCP ± SRC or HAMN with acellular mucin or LGMCP) between primary tumor and PD were approximately 18.1% in our study. In prior smaller cohorts, 3.7–15.8% of patients had discordance between primary tumor and PD.^19,22,23,39^ The observed heterogeneity in PD in HAMN may reflect heterogeneity in the primary tumor. Notably, per the 2016 PSOGI consensus, HAMNs can include “even focal high-grade dysplasia, provided it is unequivocal”; thus, this cohort represents primary tumors both with focal high-grade dysplasia and those with a predominance of high-grade dysplasia.^8^ Further characterizing HAMNs by percent of high-grade cytologic atypia would be informative in this regard but is difficult to do retrospectively in a referral center where the original operation was completed elsewhere (with specimen secondarily reviewed by pathologists here). Alternatively, such heterogeneity of metastatic disease could reflect some degree of plasticity of tumor cells in metastatic spread. A similar spectrum of disease, including HGMCP resulting from LAMNs, argues for some degree of plasticity (also reported by Reghunathan et al.,^40^ Martín-Román et al.,^22^ Choudry et al.,^39^ Memon et al.,^19^ and Rauwerdink et al.^23^); alternatively, this could represent a failure to recognize the presence of a small high-grade component in LAMN. Genomic characterization of primary LAMN and HAMN is quite limited. Most genetic analyses have been reported for peritoneal metastases arising from mucinous neoplasms, which generally report high rates of *KRAS* and *GNAS* co-mutations in LAMN, HAMN, and low-grade adenocarcinoma, with increasing rates of *TP53* and other mutations in higher-grade disease.^12,13^ We report genomic characterization of three primary LAMN and eight primary HAMN; we also report 28 metastatic deposits arising from LAMN or HAMN. We did observe one *TP53* mutation in HAMN in the absence of M1b PD. Liao et al.^12^ noted two patients with similar disease. These data suggest that this genetic alteration is not sufficient to drive peritoneal spread. Additional sequencing of primary LAMN and HAMN and paired peritoneal metastases will be critical in aiding our understanding of genetic or epigenetic transitions that drive metastasis, as well as potential progression within the primary lesion itself. These future studies should include transcriptional measurement in addition to DNA sequencing to allow for evaluation of the tumor microenvironment, which is increasingly recognized as playing a role in appendiceal cancer.^41^

For all AMNs, both RFS and OS are influenced by type of PD. Further analyses of RFS and OS stratified by both primary tumor type and type of PD are limited by low patient numbers. Although there is a trend towards decreased RFS following definitive surgery for patients with HAMN as compared with LAMN, this is likely largely secondary to increased rates of HGMCP in HAMN as compared with LAMN. OS is similar for patients with LAMN and those with HAMN. Patients with HAMN and HGMCP have the lowest OS. These patients may represent a unique biology; further analyses of this patient population in particular is warranted. It is worth noting that we observed only two recurrences when patients were without evidence of PD at time of presentation. Notably, no recurrences were seen with the spread of acellular mucin alone. This is in line with prior published studies on LAMN, including our own.^22,24,40,42–45^

Limitations of this study are those inherent to any retrospective study performed at a quaternary referral medical center. We acknowledge that this study may not capture the whole spectrum of disease secondary to referral bias. We also acknowledge potential bias in our survival analyses, with informative censoring also related to our status as a referral center with variable follow-up, especially in patients on whom we did not operate and those with extremes of disease. It should also be noted that we report OS and not disease-specific survival, which is impactful given the indolence of this disease. As an example, there were six deaths in patients with localized AMNs (five with LAMN; one with HAMN) and two deaths in those with PD limited to acellular mucin (one each with LAMN and HAMN). Only one of these deaths was related to the primary diagnosis; this patient experienced a recurrence with HGMCP. The others were attributed to additional malignancies with progressive decline in five patients, respiratory failure secondary to pneumonia in one patient, and unknown cause in one patient who received cancer care locally and only presented to MDACC for a second opinion.

Notably, up to 2021, AMNs were characterized as borderline and not malignant by the WHO, limiting reporting. We expect our population-based understanding of these tumors to improve with these changes. Although pathologic diagnoses were made by subspecialized pathologists, reviews can be limited by inconsistent sampling, ranging from representative sections to *in toto* submissions of the appendix at referring institutions. The reviews can also be limited when only selected slides are sent for review. Evaluation by a group of expert pathologists using defined criteria for tissue submission and microscopic evaluation with a focus on the extent of high-grade features and its impact on clinical behavior would be prudent. However, this would require significant effort and is beyond the scope of this study.

In summary, we present here the largest cohort of patients with HAMN reported to date. HAMNs are associated with higher rates of PD overall and higher grade of PD than LAMNs. There is a trend towards decreased RFS for HAMN as compared with LAMN; OS is not significantly different between LAMN and HAMN. Both RFS and OS are influenced by type of PD when considering all AMNs, but analyses stratifying by primary tumor type are limited by small cohorts. Our understanding of HAMNs would be further bolstered by multi-institutional collaborative efforts or population-based analyses enabled by the new classification as “malignant” by the WHO. Further molecular analysis is critical to aid our understanding of primary tumor progression and development of peritoneal metastases.

## Supplementary Information

Below is the link to the electronic supplementary material.

Supplementary file1 (DOCX 36 KB)

Supplementary file2 (DOCX 28 KB)

Supplementary file3 (DOCX 25 KB)

Supplementary file4 Figure S1. (A) Recurrence-free survival by peritoneal disease. (B) Overall survival by peritoneal disease.

Supplementary file5 Figure S2. Recurrence-free survival of LAMN with LGMCP compared to HAMN with LGMCP.

Supplementary file6 Figure S3. Recurrence-free survival of LAMN with HGMCP ± SRC compared to HAMN with HGMCP ± SRC.

Supplementary file7 Figure S4. Overall survival of LAMN with LGMCP compared to HAMN with LGMCP.

Supplementary file8 Figure S5. Overall survival of LAMN with HGMCP ± SRC compared to HAMN with HGMCP ± SRC.
