# Supplementary material for: Clinicopathologic and Molecular Characteristics of High-Grade Appendiceal Mucinous Neoplasms
Source: Ann Surg Oncol. 2025 Nov 12;33(3):2376–87. doi: 10.1245/s10434-025-18672-0 (PMC12901230; doi:10.1245/s10434-025-18672-0)
Supplement: Supplementary file 1 — Supplementary file1 (DOCX 36 KB) [file 10434_2025_18672_MOESM1_ESM.docx]

SUPPLEMENTARY TABLE 1. Published case series and cohort studies of HAMN (PSOGI classification) with a summary of pertinent results. Refer to the original publication for comprehensive results. Case reports were not included.

| Author | Year | Patients with HAMN (N) | Summary of Results |
| --- | --- | --- | --- |
| Mehta et al.^1^ | 2017 | 2 | HAMNs were grouped with adenocarcinoma for analysis. |
| Singhal et al.^2^ | 2018 | 8 | No recurrences in localized HAMN (median follow-up 72 months) or localized LAMN (median follow-up of 50 months). |
| Liao et al.^3^ | 2019 | 9 | HAMN and LAMN share high rates of KRAS and GNAS co-mutations. HAMNs uniquely had TP53 or ATM mutations. |
| Tsai et al.^4^ | 2019 | 6 | LAMNs and HAMNs shared mutations in the Wnt/β-catenin pathway (APC, RNF43, or CTNNB1 variants). |
| *Kyang et al.*^5^ | *2019* | *152** | **The authors define HAMN as “increased abundant mucinous epithelium forming glands and cytologic atypia of mucinous carcinoma in accordance with the WHO 4^th^ edition (2010) classification.”  Notably, this is not consistent with PSOGI or WHO 5^th^ edition (2019) definitions of HAMN. Thus, these are excluded.* |
| Su et al.^6^ | 2020 | 38 | Evaluated gene expression patterns of AMNs to assign subtypes as immune-enriched (IE), oncogene-enriched (OE) and mixed (M). Notable decreased OS in HAMN compared to LAMN after CRS-HIPEC. |
| Munari et al.^7^ | 2021 | 2 | KRAS mutation in 66.7% and RNF43 mutation in 66.7% of HAMNs. |
| Yanai et al.^8^ | 2021 | 8 | Compared molecular analysis of pure LAMN, HAMN, and MAC and tissue with mixed or multiple components. |
| Gonzalez et al.^9^ | 2022 | 35 | KRAS mutations in 7/35 cases and TP53 mutations in 4/35 cases. Localized HAMN had no recurrences. PD was graded according to AJCC. 2/35 cases exhibited metachronous PD (1 G1, 1 G2). Of 11/35 patients with synchronous PD (1 G1, 10 G2), 5/11 died of disease. |
| Polydorides et al.^10^ | 2022 | 15 | Limited to only localized LAMN/HAMN. Outcomes were limited to patients who had clinical follow-up for at least 6 months, which included 9/15 of HAMNs. Of those 9, there were no recurrences. All lymph nodes were negative for disease. |
| *Memon et al.^11^* | *2022* | *14** | **The actual number of HAMNs is unclear because the high-grade primary tumors (N=14) include HAMN and mucinous adenocarcinoma (i.e. AJCC G2).* |
| Halfter et al.^12^ | 2023 | 3 | HAMN grouped with LAMN for outcomes analysis. |
| Blaj et al.^13^ | 2023 | 15 | Of patients with PMP who underwent CRS-HIPEC, LAMN (vs HAMN) showed improved OS (106.45 vs. 61.15 months, *p* < 0.0001) and PFS (90.63 vs. 40.52 months, p < 0.0001). |
| Martín-Román et al.^14^ | 2023 | 5 | Of 5 HAMNs, the PD was 1 AM, 1 LGMCP, and 3 HGMCP. Primary appendiceal tumor classification was not significant on multi-variate analysis of DFS and OS. |
| Rauwerdrink et al.^15^ | 2024 | 8 | 13.3% discordance rate between primary tumor histology and PMP grade, while PMP grade strongly influences survival. |
| Dartigues et al.^16^ | 2025 | 34 | Limited to patients who underwent RHC. No LN metastases were identified within HAMN. In patients with perforation, LAMN had improved OS and PFS than HAMN. HAMN/LAMN without perforation had a similar prognosis. HAMN had a higher rate of HGMCP and HGMCP + SRCs than LAMN. |

1. Mehta A, Mittal R, Chandrakumaran K, et al. Peritoneal Involvement Is More Common Than Nodal Involvement in Patients With High-Grade Appendix Tumors Who Are Undergoing Prophylactic Cytoreductive Surgery and Hyperthermic Intraperitoneal Chemotherapy. *Dis Colon Rectum*. 2017;60(11):1155-1161. doi:10.1097/dcr.0000000000000869

2. Singhal S, Giner-Segura F, Barnes TG, Hompes R, Guy R, Wang LM. The value of grading dysplasia in appendiceal mucinous neoplasm in the absence of pseudomyxoma peritonei. *Histopathology*. 2018;73(2):351-354. doi:<https://doi.org/10.1111/his.13518>

3. Liao X, Vavinskaya V, Sun K, et al. Mutation profile of high-grade appendiceal mucinous neoplasm. *Histopathology*. Feb 2020;76(3):461-469. doi:10.1111/his.13986

4. Tsai JH, Yang CY, Yuan RH, Jeng YM. Correlation of molecular and morphological features of appendiceal epithelial neoplasms. *Histopathology*. Oct 2019;75(4):468-477. doi:10.1111/his.13924

5. Kyang LS, Alzahrani NA, Alshahrani MS, Rahman MK, Liauw W, Morris DL. Early recurrence in peritoneal metastasis of appendiceal neoplasm: Survival and prognostic factors. *European Journal of Surgical Oncology*. 2019/12/01/ 2019;45(12):2392-2397. doi:<https://doi.org/10.1016/j.ejso.2019.06.015>

6. Su J, Jin G, Votanopoulos KI, et al. Prognostic Molecular Classification of Appendiceal Mucinous Neoplasms Treated with Cytoreductive Surgery and Hyperthermic Intraperitoneal Chemotherapy. *Annals of Surgical Oncology*. 2020/05/01 2020;27(5):1439-1447. doi:10.1245/s10434-020-08210-5

7. Munari G, Businello G, Mattiolo P, et al. Molecular profiling of appendiceal serrated lesions, polyps and mucinous neoplasms: a single-centre experience. *Journal of Cancer Research and Clinical Oncology*. 2021/07/01 2021;147(7):1897-1904. doi:10.1007/s00432-021-03589-4

8. Yanai Y, Saito T, Hayashi T, et al. Molecular and clinicopathological features of appendiceal mucinous neoplasms. *Virchows Archiv*. 2021/03/01 2021;478(3):413-426. doi:10.1007/s00428-020-02906-5

9. Gonzalez RS, Carr NJ, Liao H, Pai RK, Agostini-Vulaj D, Misdraji J. High-Grade Appendiceal Mucinous Neoplasm: Clinicopathologic Findings in 35 Cases. *Arch Pathol Lab Med*. Dec 1 2022;146(12):1471-1478. doi:10.5858/arpa.2021-0430-OA

10. Polydorides AD, Wen X. Clinicopathologic parameters and outcomes of mucinous neoplasms confined to the appendix: a benign entity with excellent prognosis. *Modern Pathology*. 2022/11/01/ 2022;35(11):1732-1739. doi:<https://doi.org/10.1038/s41379-022-01114-7>

11. Memon AA, Godbole C, Cecil T, et al. Overall Survival is More Closely Associated with Peritoneal than Primary Appendiceal Pathological Grade in Pseudomyxoma Peritonei with Discordant Pathology. *Ann Surg Oncol*. Apr 2022;29(4):2607-2613. doi:10.1245/s10434-021-10994-z

12. Halfter K, Schubert-Fritschle G, Klauschen F, et al. The other colon cancer: a population-based cohort study of appendix tumour trends and prognosis. *Colorectal Dis*. May 2023;25(5):943-953. doi:10.1111/codi.16510

13. Blaj S, Dora D, Lohinai Z, et al. Prognostic Factors in Pseudomyxoma Peritonei with Emphasis on the Predictive Role of Peritoneal Cancer Index and Tumor Markers. *Cancers (Basel)*. Feb 19 2023;15(4)doi:10.3390/cancers15041326

14. Martín-Román L, Hannan E, Faraz Khan M, et al. Correlation between PSOGI pathological classification and survival outcomes of patients with pseudomyxoma peritonei treated using cytoreductive surgery and HIPEC: national referral centre experience and literature review. *Pleura Peritoneum*. Jun 2023;8(2):65-74. doi:10.1515/pp-2023-0001

15. Rauwerdink P, Al-Toma D, Wassenaar ECE, et al. Reclassification of Appendiceal Mucinous Neoplasms and Associated Pseudomyxoma Peritonei According to the Peritoneal Surface Oncology Group International Consensus: Clinicopathological Reflections of a Two-Center Cohort Study. *Ann Surg Oncol*. Dec 2024;31(13):8572-8584. doi:10.1245/s10434-024-16254-0

16. Dartigues P, Kepenekian V, Illac-Vauquelin C, et al. Insights into the Clinical Prognosis of High-grade Appendiceal Mucinous Neoplasms. *The American Journal of Surgical Pathology*. 2025:10.1097/PAS.0000000000002373. doi:10.1097/pas.0000000000002373
