# Supplementary material for: Clinicopathologic and Molecular Characteristics of High-Grade Appendiceal Mucinous Neoplasms
Source: Ann Surg Oncol. 2025 Nov 12;33(3):2376–87. doi: 10.1245/s10434-025-18672-0 (PMC12901230; doi:10.1245/s10434-025-18672-0)
Supplement: Supplementary file 2 — Supplementary file2 (DOCX 28 KB) [file 10434_2025_18672_MOESM2_ESM.docx]

| Clinical Practice Guideline | Year | Notes |
| --- | --- | --- |
| Chicago Consensus Guidelines^1^ | 2018 | No discrete recommendations. Under the appendiceal adenocarcinoma management pathway, patients without metastatic disease undergo RHC, with an asterisk denoting that HAMNs and well-differentiated mucinous adenocarcinoma may be treated similar to low-grade lesions, meaning that margin-negative appendectomy is sufficient.  Updated recommendations by the PSM Consortium Group (previously the Chicago Consensus) are in pre-print status.^2^ |
| PSOGI/ EURACAN^3^ | 2021 | Consensus Statements (Delphi technique, GRADE-based)   1. Non-perforated HAMN (<T4, M0) without residual disease, consider adjuvant RHC [67%, 2C] 2. Non-perforated HAMN (<T4, M0) without residual disease, consider adjuvant CRS-HIPEC with RHC [56.4%, 2C] 3. Perforated HAMN (<T4, M0) without residual disease, an adjuvant CRS-HIPEC with RHC should always be performed [58%, 1B] 4. HAMN with M1b PD, CRS-HIPEC with RHC should always be performed [76%, 2B] |
| Italian Society of Surgical Oncology^4^ | 2023 | Consensus Statements (RAM method):   1. Non-perforated HAMN after appendectomy, RHC should be considered (AMS 9 [appropriate], IPR 9-9 [valid]) 2. Perforated HAMN and/or peritoneal disease are found after appendectomy, CRS-HIPEC with RHC should be considered (AMS 9 [appropriate], IPR 9-9 [valid]) |
| ASCRS^5^ | 2025 | Consensus Statements (GRADE-based):   1. Systemic chemotherapy may improve survival in patients with metastatic, lymph node positive, HAMN, and adenocarcinoma with peritoneal metastases. Routine use of systemic chemotherapy for LAMNs or well-differentiated mucinous adenocarcinoma with peritoneal spread is not recommended [1C]   Authors note that HAMNs without perforation or peritoneal involvement, and a negative microscopic margin when found incidentally in an appendectomy specimen, can generally be treated with appendectomy alone. However, care should be taken to exclude the presence of associated invasive adenocarcinoma, including comprehensive histologic evaluation of the entire surgical specimen by an expert pathologist. Given the rarity and paucity of data on HAMNs, there should be a low threshold to review and discuss these cases in a multidisciplinary setting. |
| AJCC 9^th^ version^6^ | 2024 | RHC is recommended in some instances after appendectomy (pT1-T4a). For localized LAMNs, an appendectomy represents definitive surgical treatment and a RHC is not recommended. The Tis(LAMN) category does not apply to HAMNs; the latter are staged using the appendiceal adenocarcinoma T categories. |
| NCCN Guidelines^©^ Version 2.2025^7^ | 2025 | Tis(LAMN)- and T1-staged tumors may be managed with appendectomy alone if negative margins are obtained during resection and there is no evidence of angiolymphatic invasion. T1 and T2 tumors with unfavorable characteristics such as angiolymphatic invasion or positive margins should be considered for RHC (discussion update in progress). |

SUPPLEMENTARY TABLE 2. Clinical practice guidelines provided by surgical societies.

*PSM* Peritoneal Surface Malignancies, *HAMN* high-grade appendiceal mucinous neoplasm, *LAMN* low-grade appendiceal mucinous neoplasm, *RHC* right hemicolectomy, *PSOGI* Peritoneal Surface Group Oncology International, *EURACAN* European Reference Network for Rare Adult Solid Cancers, *GRADE* Grades of Recommendation, Assessment, Development, and Evaluation, *RAM* RAND/UCLA Appropriateness Method, *AMS* appropriateness median score, *IPR* interpercentile range, *ASCRS* American Society of Colon and Rectal Surgeons, *AJCC* American Joint Committee on Cancer, *NCCN^©^* National Comprehensive Cancer Network^©^, *NCCN Guidelines^©^* NCCN Clinical Practice Guidelines in Oncology

1. The Chicago Consensus on peritoneal surface malignancies: Management of appendiceal neoplasms. *Cancer*. Jun 1 2020;126(11):2525-2533. doi:10.1002/cncr.32881

2. Group PATW, Group PC, Turaga KK. Consensus guideline for the management of patients with appendiceal tumors Part 1: Appendiceal tumors without peritoneal involvement. *medRxiv*. 2024:2024.04.09.24305468. doi:10.1101/2024.04.09.24305468

3. Govaerts K, Lurvink RJ, De Hingh IHJT, et al. Appendiceal tumours and pseudomyxoma peritonei: Literature review with PSOGI/EURACAN clinical practice guidelines for diagnosis and treatment. *European Journal of Surgical Oncology*. 2021/01/01/ 2021;47(1):11-35. doi:<https://doi.org/10.1016/j.ejso.2020.02.012>

4. Vaira M, Robella M, Guaglio M, et al. Diagnostic and Therapeutic Algorithm for Appendiceal Tumors and Pseudomyxoma Peritonei: A Consensus of the Peritoneal Malignancies Oncoteam of the Italian Society of Surgical Oncology (SICO). *Cancers (Basel)*. Jan 24 2023;15(3)doi:10.3390/cancers15030728

5. Gaertner WB, Brown S, Deutsch M, et al. The American Society of Colon and Rectal Surgeons Clinical Practice Guidelines for the Management of Appendiceal Neoplasms. *Dis Colon Rectum*. 2025:10.1097/DCR.0000000000003796. doi:10.1097/dcr.0000000000003796

6. Hanna N, Hanna AN, Hanna DN. AJCC Cancer Staging System Version 9: Appendiceal Adenocarcinoma. *Annals of Surgical Oncology*. 2024/04/01 2024;31(4):2177-2180. doi:10.1245/s10434-024-14892-y

7. Referenced with permission from the NCCN Clinical Practice Guidelines in Oncology (NCCN Guidelines®) for Colon Cancer V.2.2025. © National Comprehensive Cancer Network, Inc. 2025. All rights reserved. Accessed [May 12, 2025]. To view the most recent and complete version of the guideline, go online to NCCN.org.
