# Supplementary material for: Clinicopathologic and Molecular Characteristics of High-Grade Appendiceal Mucinous Neoplasms
Source: Ann Surg Oncol. 2025 Nov 12;33(3):2376–87. doi: 10.1245/s10434-025-18672-0 (PMC12901230; doi:10.1245/s10434-025-18672-0)
Supplement: Supplementary file 3 — Supplementary file3 (DOCX 25 KB) [file 10434_2025_18672_MOESM3_ESM.docx]

SUPPLEMENTARY TABLE 3. Amino acid and protein changes for each somatic variant detected, classified by primary and peritoneal tissue types.

| GENE | LAMN-LGMCP | LAMN-HGMCP | HAMN-AM | HAMN-LGMCP | HAMN-HGMCP |
| --- | --- | --- | --- | --- | --- |
| Sample (N) | 14 | 4 | 2 | 6^b^ | 8 |
| KRAS | G12A  G12D^a^ (9)  G12V (5)  G13D (2) | G12C  G12D  G13dup  G12V | G12D  G12V | G12D (2)  G12V  Q61H | G12C  G12D (5)  G12V  G13D  Q61H |
| GNAS | R201C (3)  R201H^a^ (11) | R201C  R201H | R201H | R201C  R201H (3) | R201C (4)  R201H (3) |
| TP53 |  |  | G245S | R175H (2) | P278R  Y220C  V272E |
| SMAD2 | S464* (2)  P305L |  |  |  | S464*  R74Q |
| SMAD4 | R496H  W524C  R445*  D415fs*  D424V  Q366E  R361H |  |  |  | R361H |
| ARID1B | Q1236fs*16  S1174* |  |  |  | S361L |
| RNF43 | K45* |  | R113* |  | I209M |
| SMAD3 | R268H  ? |  |  |  | R74Q |
| APC | A1366S |  |  |  | G1412* |
| CTNNB1 |  |  | S33F  S37A | G34R |  |
| FBXW7 | R505C | G422A |  |  |  |
| RBM10^c^ | X |  | X |  |  |
| Additional variants (N=1, 3%) | SMAD3 CCND2 ACVR2A  IGF1  PRG4  CXCR4 EWSR1  ASXL1  FUBP1  LRP1B  EPHA3  MED12  MGA  CDKN2A  EGFR  MST1R  NOTCH2  PTPRT  TSC2  WRN  LRPB1  MAP2K4 | ASCC3  PIK3CA  ATM | CUX1  HERC2  MTOR  NCOR1  PLCG2  ARID1A  DAXX  DDR2  KEL  NTRK3  SOX9 | ARID2  CUL3  DNMT3A  GRIN2A  RAD50  SF3B1  TET2 | CDK12  IDH1  PMS2  PTPRD  SMARCB1  TAP2  TSC2  TGFBR2 |

^a^Source tissue: primary LAMN

^b^1 sample did not have any variants

^c^Germline mutation
