# Supplementary figures and images for: Clinicopathologic and Molecular Characteristics of High-Grade Appendiceal Mucinous Neoplasms
Source: Ann Surg Oncol. 2025 Nov 12;33(3):2376–87. doi: 10.1245/s10434-025-18672-0 (PMC12901230; doi:10.1245/s10434-025-18672-0)

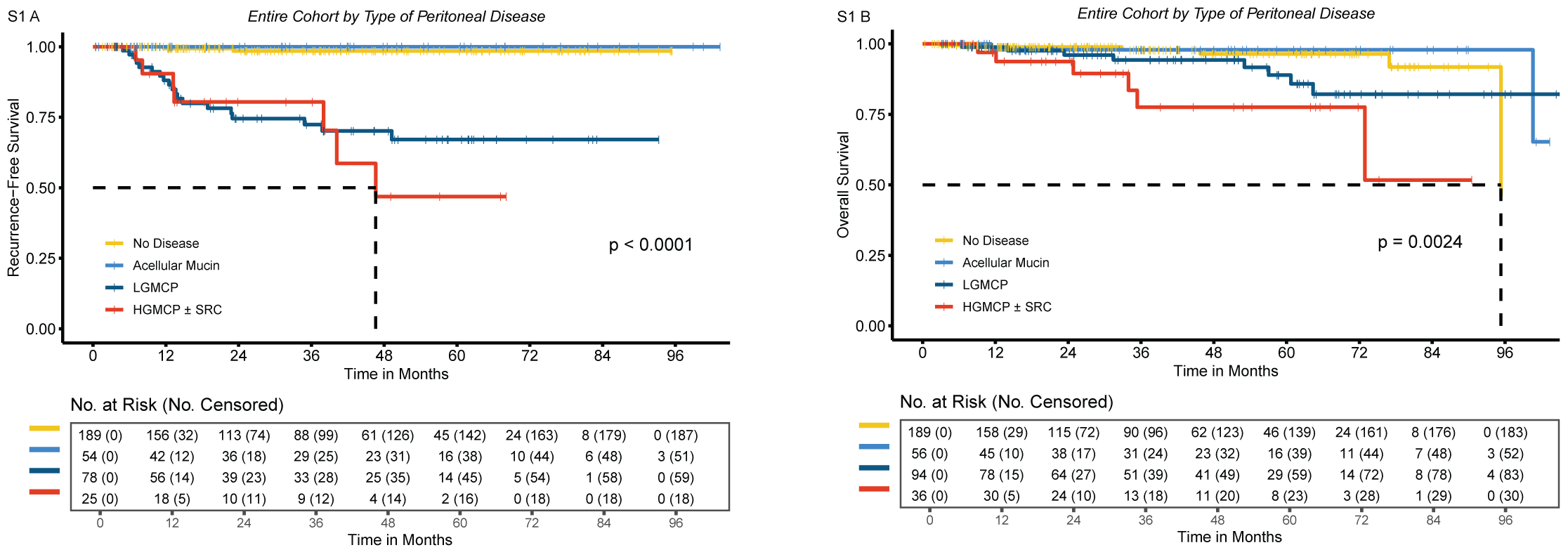

Supplement: Supplementary file 4 — Supplementary file4 Figure S1. (A) Recurrence-free survival by peritoneal disease. (B) Overall survival by peritoneal disease. [file 10434_2025_18672_MOESM4_ESM.tif]

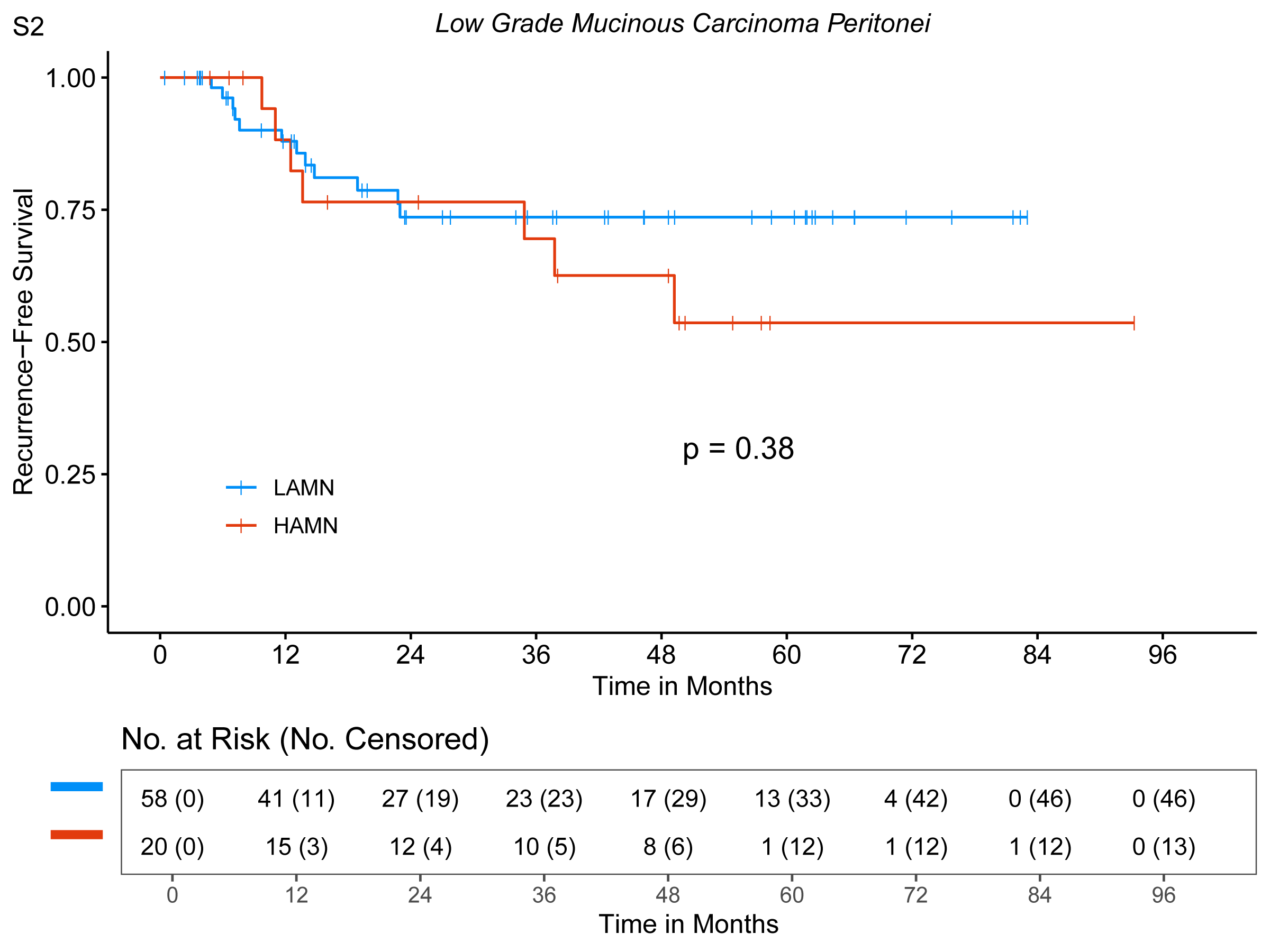

Supplement: Supplementary file 5 — Supplementary file5 Figure S2. Recurrence-free survival of LAMN with LGMCP compared to HAMN with LGMCP. [file 10434_2025_18672_MOESM5_ESM.tif]

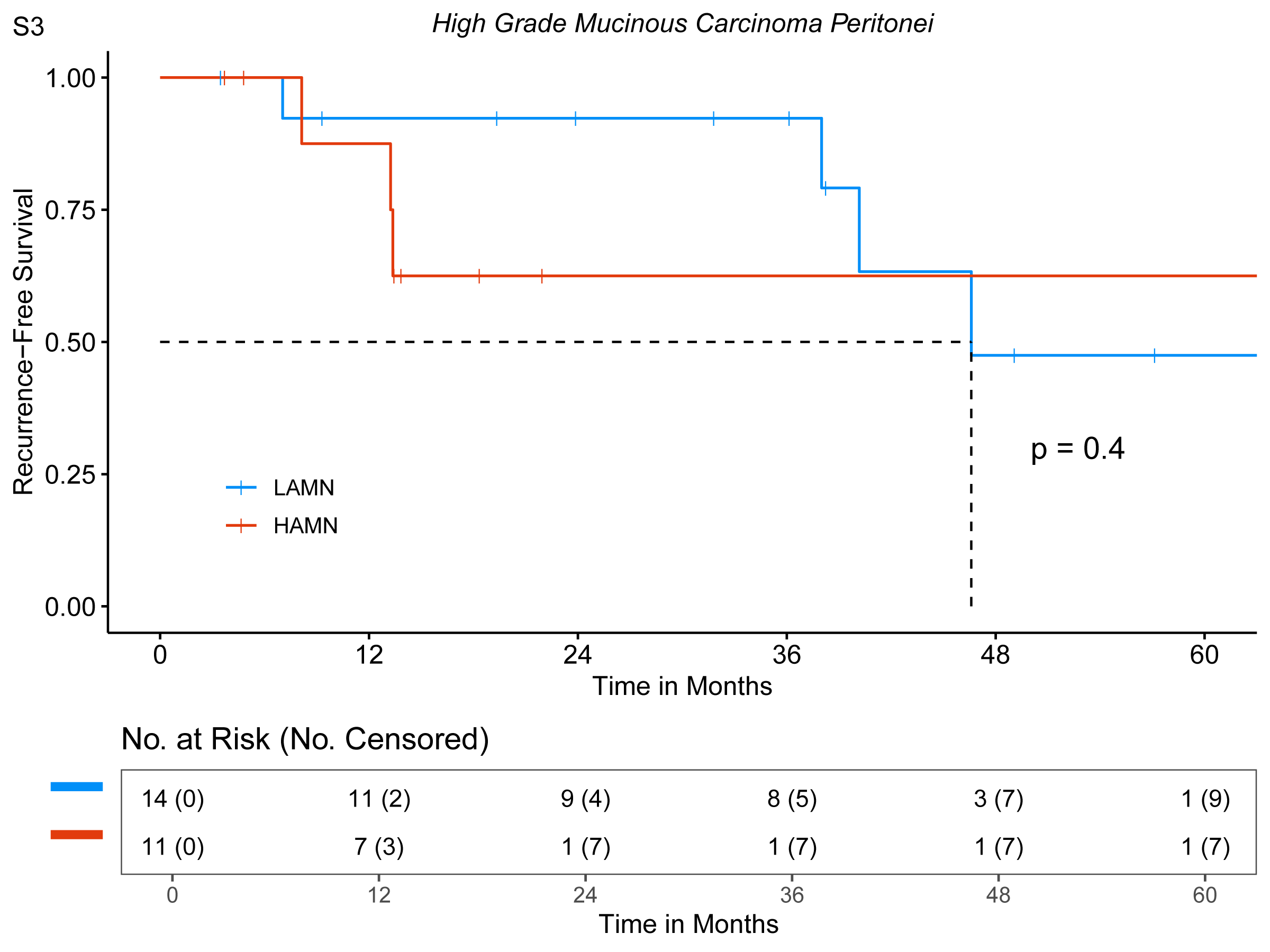

Supplement: Supplementary file 6 — Supplementary file6 Figure S3. Recurrence-free survival of LAMN with HGMCP ± SRC compared to HAMN with HGMCP ± SRC. [file 10434_2025_18672_MOESM6_ESM.tif]

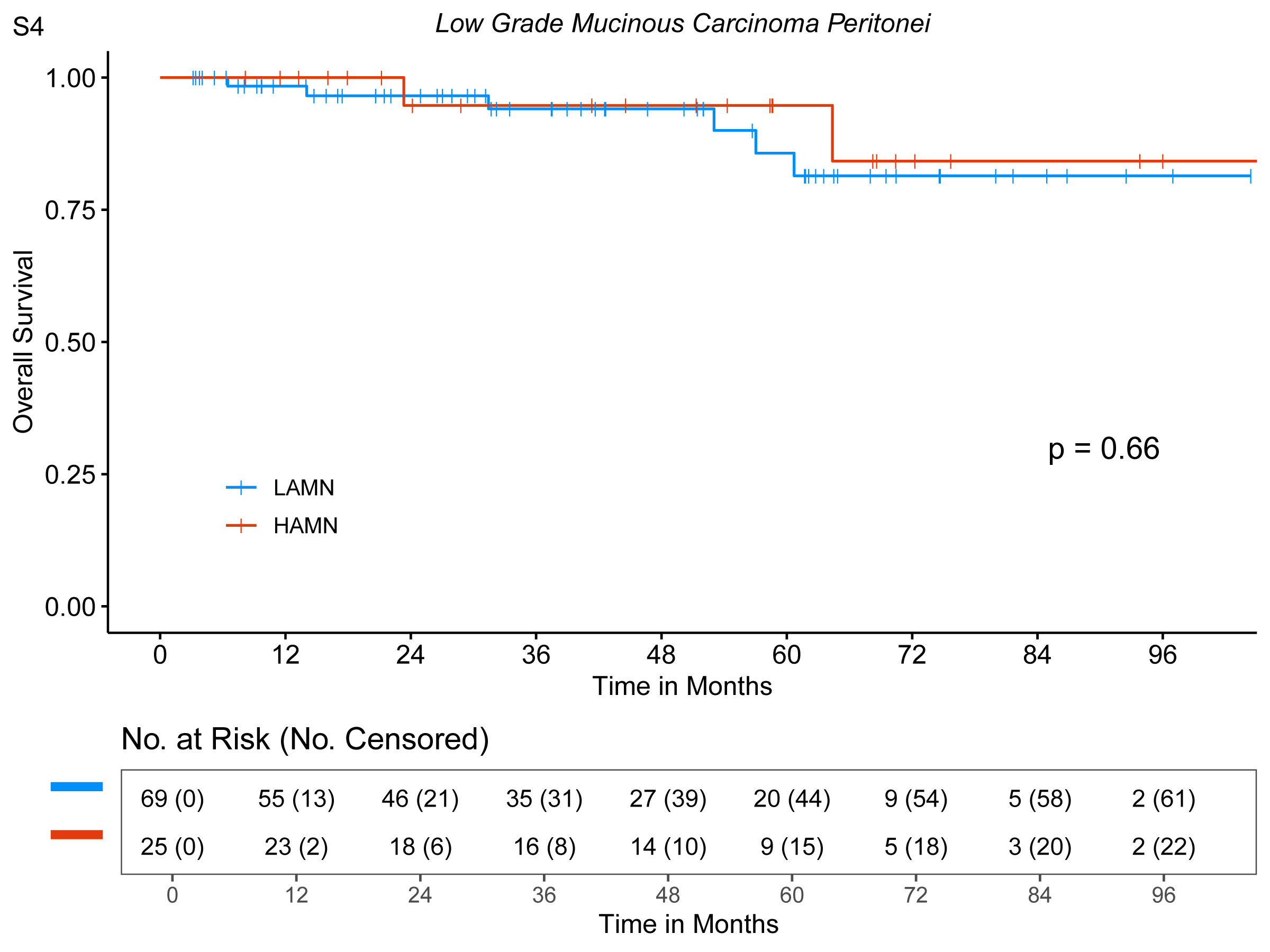

Supplement: Supplementary file 7 — Supplementary file7 Figure S4. Overall survival of LAMN with LGMCP compared to HAMN with LGMCP. [file 10434_2025_18672_MOESM7_ESM.tif]

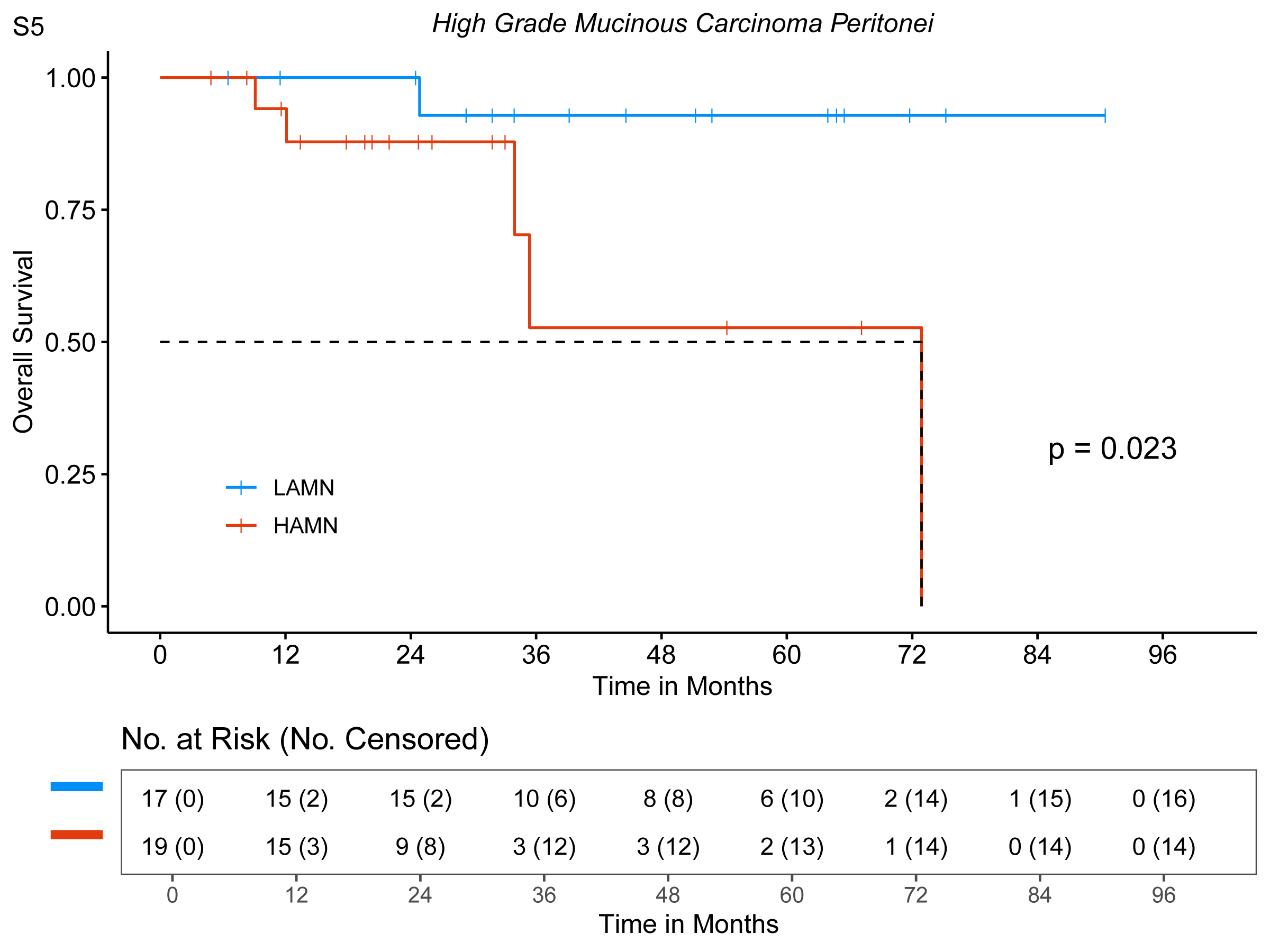

Supplement: Supplementary file 8 — Supplementary file8 Figure S5. Overall survival of LAMN with HGMCP ± SRC compared to HAMN with HGMCP ± SRC. [file 10434_2025_18672_MOESM8_ESM.tif]
